# In-vitro activity of the novel β-lactam/β-lactamase inhibitor combinations and cefiderocol against carbapenem-resistant *Pseudomonas* spp. clinical isolates collected in Switzerland in 2022

**DOI:** 10.1007/s10096-024-04994-6

**Published:** 2024-12-20

**Authors:** Christophe Le Terrier, Maxime Bouvier, Auriane Kerbol, Chloé Dell’Acqua, Nara Network members, Patrice Nordmann, Laurent Poirel

**Affiliations:** 1 Medical and Molecular Microbiology Unit, Department of Medicine, Faculty of Science and Medicine, University of Fribourg, Chemin du Musée 18, Fribourg, CH-1700 Switzerland; 2 Swiss National Reference Center for Emerging Antibiotic Resistance (NARA), University of Fribourg, Fribourg, Switzerland; 3 Division of Intensive Care Unit, University Hospitals of Geneva, Geneva, Switzerland; 4 Emergency Department, Fribourg Hospitals, Fribourg, Switzerland

**Keywords:** Cefiderocol, Ceftolozane, Imipenem, Meropenem, Avibactam, Relebactam, Vaborbactam, Β-lactamase, Carbapenemase

## Abstract

To evaluate the in-vitro activity of the novel commercially-available drugs, including meropenem-vaborbactam (MEV), ceftazidime-avibactam (CZA), ceftolozane-tazobactam (C/T), imipenem-relebactam (IPR) as well as cefiderocol (FDC), against carbapenem-resistant *Pseudomonas* spp. (CRP) isolates. All CRP isolates collected at the Swiss National Reference Laboratory (NARA) over the year 2022 (*n* = 170) have been included. Most of these isolates (*n* = 121) were non-carbapenemase producers. Among the 49 carbapenemase producers, 47 isolates produced metallo-β-lactamases (MBL) including NDM-1 (*n* = 11), VIM-like (*n* = 28), IMP-like (*n* = 7), and both NDM-1 and VIM-2 (*n* = 1) and two isolates produced the class A carbapenemase GES-5. Susceptibility testing was determined by broth microdilution method (BMD), or disk diffusion test, and results interpreted following EUCAST guidelines. The susceptibility rates for MEV, CZA, C/T and IPR were found to be 41%, 45%, 59% and 58%, respectively, for the whole set of isolates tested. Among non-carbapenemase producers, susceptibility rates for these β-lactam/β-lactamase inhibitors (BL/BLI) combinations were higher, determined at 55%, 61%, 83%, and 82%, respectively. The overall susceptibility of carbapenemase-producing *Pseudomonas* spp. to novel BL/BLI was relatively low, while 80% of these isolates demonstrated susceptibility to FDC, with a similar proportion (79%) observed among MBL producers. A total of 10 MBL-producing isolates (6%), mainly NDM-1, were found to exhibit resistance to all drugs tested, with the exception of colistin. FDC exhibited an excellent in-vitro activity against this collection of CRP recovered from Switzerland in 2022, including MBL producers. The new BL/BLI combinations displayed significant activity against non-carbapenemase CRP, with IPR and C/T showing the highest susceptibility rates.

**Supplementary Information:**

The online version contains supplementary material available at 10.1007/s10096-024-04994-6.

## Introduction

The global spread of Gram-negative bacteria exhibiting multidrug- or even pandrug resistance is a worrying concern [1]. In 2017, the World Health Organization (WHO) ranked carbapenem-resistant *Pseudomonas aeruginosa* (CPRA) as well as carbapenem-resistant *Enterobacterales* and carbapenem-resistant *Acinetobacter baumannii* in the critical global priority list of pathogens. This ranking led to numerous research and development projects on antibiotic resistance, as well as the development of new antibiotics and β-lactamase inhibitors [2]. *P. aeruginosa* is an opportunistic Gram-negative pathogen widely distributed in the environment but also in hospitals [3, 4]. Multidrug resistance is commonly observed with this nosocomial pathogen which possesses the ability to rapidly adapt to antibiotics and develop combined resistance mechanisms through mutations. Hence, related severe infections, particularly in immunocompromised patients, are extremely difficult to treat [3–5].

Intrinsic resistance in that species is partly due to low permeability of the outer membrane, expression of efflux systems (MexAB-OprM, MexCD-OprJ, MexXY and MexEF-OprN), and production of chromosomally-encoded β-lactamases, namely PDC- and OXA-50-like enzymes [3–6]. Acquired resistance to carbapenems is mainly related to combinations of non-enzymatic mechanisms like low expression of porin-encoding genes, mutations or truncations in chromosomal porin OprD genes, overexpression of genes encoding efflux pumps, associated with overexpression of chromosomal β-lactamase genes [6–9]. On the other hand, acquired resistance to carbapenems may be related to the production of acquired carbapenemases, mostly belonging to Ambler class B (i.e. NDM-, VIM-, IMP-type MBLs), or class A (GES-type enzymes). Resistance to the siderophore cephalosporin cefiderocol (FDC) in that species has also been recently related to multiples factors, including mutations in the genes encoding TonB iron transporters [10–15]. Despite the meropenem-vaborbactam (MEV) combination has only been approved by the EMA but not the FDA for this indication [16, 17], a series of novel β-lactam/β-lactamase inhibitors (BL/BLI) combinations, including ceftazidime-avibactam (CZA), ceftolozane-tazobactam (C/T) and imipenem-relebactam (IPR), can now be considered for the treatment of carbapenem-resistant *Pseudomonas* spp. associated infections. All of these BL/BLIs basically constitute interesting therapeutical options against multidrug-resistant *P. aeruginosa* isolates, although they remain inefficient against MBL producers [18, 19].

Besides these novel BL/BLI combos, FDC was approved in 2020 for the treatment of infections associated to carbapenem-resistant Gram-negative bacteria, including carbapenem-resistant *Pseudomonas* spp. (CRP), since this antibiotic is not significatively hydrolyzed by most carbapenemases, including MBLs [20, 21].

The objective of our study was therefore to assess the in-vitro activity of these five recently developed and approved therapeutic alternatives in Europe (MEV, CZA, C/T, IPR and FDC) against carbapenem-resistant *Pseudomonas* spp. clinical isolates currently circulating in Switzerland in 2022.

## Materials and methods

### Bacterial isolates

All CRP (n *=* 170) clinical isolates collected and characterized during the year 2022 at the Swiss National Reference Center for Emerging Antibiotic Resistance (NARA) and recovered across all Switzerland were included in this study. On a daily basis, all laboratories in Switzerland are requested to submit their CRP isolates to NARA for further analysis. Only one isolate per patient was included in the collection. Most of the isolates were *P. aeruginosa* (*n* = 161), but this collection also included other species such as *Pseudomonas citronellolis* (*n* = 3), *Pseudomonas putida* (*n* = 2), *Pseudomonas fluorescens* (*n* = 1), *Pseudomonas alcaligenes* (*n* = 2) and *Pseudomonas nitroreducens* (*n* = 1). Those isolates produced different resistance mechanisms such as carbapenemases (*n* = 49), among which there were producers of Ambler class B β-lactamases (*n* = 47) such as NDM-1 (*n* = 11), VIM-1 (*n* = 2), VIM-2 (*n* = 20), VIM-4 (*n* = 4), VIM-5 (*n* = 2), IMP-1 (*n* = 5), IMP-7 (*n* = 1), IMP-13 (*n* = 1) or both NDM-1 and VIM-2 (*n* = 1), and producers of the Ambler class A carbapenemase GES-5 (*n* = 2).

All isolates collected were tested for carbapenemase production by using the RAPIDEC^®^ Carba NP test [22]. In case of positivity, the immunochromatographic NG-Test^®^ CARBA-5 test was subsequently used to identify the specific carbapenemase type [23], followed by confirmation using PCR and sequencing. In case of a negative RAPIDECⓇ Carba NP test, a solid antibiogram was performed using a Mueller-Hinton agar plate supplemented with cloxacillin 2000 mg/L, in order to evidence a putative AmpC overproduction, as evidenced by the notable restoration of susceptibility to ceftazidime and imipenem observed when performing solid Mueller-Hinton agar-based antibiograms using plates supplemented with cloxacillin 2000 mg/L. Thus, our collection of CRP was composed of carbapenemase-producing and non-carbapenemase-producing isolates.

### Susceptibility testing methods for BL/BLI combos, FDC and colistin

Categorization was performed using the disk diffusion method on Mueller-Hinton agar (MH-agar, Bio-Rad Laboratories, Marnes la Coquette, France) for the four BL/BLI combinations, using disks MEV30 (ref MEV30C, Mast Group, Reinfeld, Germany), CZA14 (ref 12008071, Bio-Rad), IPR35 (ref IMR35C, Mast Group) and C/T40 (ref 68040, Bio-Rad), following EUCAST 2024 Guidelines [24].

Interpretation was based on EUCAST breakpoints considering the resistant category as the diameter of disk inhibition for MEV < 14 mm, IPR < 22 mm, CZA < 17 mm (with an area of technical uncertainty between 16 mm and 17 mm), and C/T < 23 mm [25]. To enhance accuracy, and mitigate technical uncertainty, MIC values were determined in duplicate by broth microdilution method using Mueller-Hinton broth (Bio-Rad) for all strains showing a diameter within +/- 3 mm of the EUCAST clinical breakpoints using the disk diffusion method [25]. The clinically-used BL/BLI combinations MEV, CZA, C/T and IPR were therefore evaluated by broth microdilution (BMD) using a fixed concentration of 4 mg/L for avibactam (HY-14879), tazobactam (HY-W009168) and relebactam (HY-16752), and 8 mg/L for vaborbactam (HY-19930) purchased from MedChem Express (Luzern, Switzerland) [18, 19, 25–27]. Ceftazidime and ceftolozane were purchased from Sigma-Aldrich (Saint-Louis, USA), while imipenem and meropenem were from HuiChem (Shanghai, China). MIC values were interpreted based on EUCAST 2024 breakpoints, defining resistant isolate when MIC values > 8 mg/L for CZA, > 4 mg/L for C/T, > 8 mg/L for MEV and > 2 mg/L for IPR [25]. To determine FDC susceptibility, MIC values were determined in duplicate by only BMD using the commercial UMIC-test^®^ method (Brucker, Germany) following guidelines and reading guide from EUCAST [24]. In the event of a discrepancy between the two MIC results, a triplicate was conducted to assess the accurate MIC value. Interpretation was based on EUCAST 2024 breakpoints categorizing resistant isolates for those showing MIC values of FDC > 2 mg/L [25]. The susceptibility testing of colistin by BMD using colistin tablet 0.8 mg from ADATAB^®^ Mast Group (Reinfeld, Germany) was also determined for comparison. Reference strains *Escherichia coli* ATCC 25922, *E. coli* ATCC 35218, *Klebsiella pneumoniae* ATCC 700603, *K. pneumoniae* ATCC BAA-2814, and *P. aeruginosa* ATCC 27853 were used as quality control strains for all antimicrobial agents evaluated according to EUCAST [28].

### Whole-sequencing analysis (WGS)

WGS was conducted on all isolates exhibiting resistance to FDC in order to elucidate the underlying molecular mechanisms of this resistance pattern. To ensure accurate comparison, WGS was also conducted on an equivalent number of FDC-susceptible isolates randomly selected from the collection. The entire genome was sequenced using a MiSeq Illumina platform (Illumina, San Diego, CA, USA) using the Nextera sample preparation method with 2 × 150 bp paired end reads. Illumina short reads were assembled using Shovill pipeline from Galaxy tools (http://usegalaxy.org). Sequence types, the presence of resistance genes, and speciation were confirmed, using MLST version 2.0, ResFinder version 4.1 [29], and KmerFinder version 3.2 [30], on the Center for Genomic Epidemiology platform (https://cge.cbs.dtu.dk); contigs were generated and annotated using Prokka [31]. Alignment for specific proteins sequences associated to FDC resistance was performed using Multialin sequence alignment (http://multalin.toulouse.inra.fr/multalin) [32] using *P. aeruginosa* PAO1 (GenBank accession no. NC_002516) as the reference sequence. Sequences data from this study was submitted to the National Center for Biotechnology Information’s Sequence Read Archive (BioProject no. PRJNA1167923).

## Results

### Susceptibility to the newly developed BL/BLI combinations against CRP clinical isolates

Susceptibility rates of 41%, 45%, 59%, 58% were found for MEV, CZA, C/T and IPR, respectively, when testing all isolates (Table 1). When considering only non-carbapenemase producers (*n* = 121), susceptibility rates for these BL/BLI combos were higher, namely at 55%, 61%, 83% and 82% for MEV, CZA, C/T and IPR, respectively, highlighting that C/T and IPR were the most effective BL/BLI combinations against this subgroup of isolates. However, when testing carbapenemase producers only (*n* = 49), including 47 MBL- and two GES-5-producers, only two isolates (VIM-2 producers) were found to be susceptible to MEV (4%), those isolates being actually susceptible to meropenem alone. Only two (4%) and one (2%) isolate were found to be susceptible to CZA and C/T, respectively, those isolates being GES-5 producers. Noteworthy, none of the carbapenemase-producing isolates showed susceptibility to IPR. Interestingly, C/T was the most effective BL/BLI options against MEV-resistant and IPR-resistant isolates, while IPR was the best BL/BLI agent against CZA-resistant, C/T-resistant and FDC-resistant isolates.

**Table 1:** Evaluation of novel drug combinations against multidrug-resistant *Pseudomonas* spp. isolates

Carbapenem-resistant Pseudomonas spp.		% of susceptible isolates^a, b^					
		BL/BLI combinations				Cefiderocol	Colistin
		MEV	CZA	C/T	IPR	FDC	COL
All *n* = 170		41%	45%	59%	58%	91%	96%
Carbapenemase-producing *Pseudomonas* spp. *n* = 49		4%	4%	2%	0%	80%	98%
MBL-producing *Pseudomonas* spp. *n* = 47		4%	0%	0%	0%	79%	98%
NDM- producing *Pseudomonas* spp. *n* = 12		0%	0%	0%	0%	33%	100%
VIM- producing *Pseudomonas* spp. *n* = 29		7%	0%	0%	0%	93%	97%
IMP-producing *Pseudomonas* spp. *n* = 7		0%	0%	0%	0%	100%	100%
GES-5-producing *Pseudomonas* spp. *n* = 2		0%	100%	50%	0%	100%	100%
Non-carbapenemase-producing *Pseudomonas* spp. *n* = 121		55%	61%	83%	82%	95%	95%
MEV-resistant *Pseudomonas* spp. *n* = 101		0%	19%	38%	35%	86%	96%
CZA-resistant *Pseudomonas* spp. *n* = 94		13%	0%	29%	37%	85%	97%
C/T-resistant *Pseudomonas* spp. *n* = 69		9%	3%	0%	20%	81%	97%
IPR-resistant *Pseudomonas* spp. *n* = 71		7%	17%	23%	0%	86%	97%
FDC-resistant *Pseudomonas* spp. *n* = 16		13%	13%	19%	38%	0%	100%
COL-resistant *Pseudomonas* spp. *n* = 7		43%	57%	71%	71%	100%	0%

^a^ According to EUCAST

^b^Antibiotic abbreviations; MEV, meropenem/vaborbactam; CZA, ceftazidime-avibactam C/T, ceftolozane-tazobactam; IPR, imipenem/relebactam,; FDC, cefiderocol; COL, colistin. The concentration of ß-lactamase inhibitors was fixed at 4 mg/L for avibactam, tazobactam, relebactam, except for vaborbactam which was fixed at 8 mg/L

Analysis of a set of non-carbapenemase and C/T-resistant isolates revealed that twelve isolates produced an ESBL, including PER-like, VEB-like, BEL-like, and GES-like enzymes, as detailed in the Supplementary Table. Furthermore, two strains were identified as *P. alcaligenes*, a species naturally producing the class B3 MBL PAM-1, and one strain was identified as *P. putida*. The analysis of AmpC amino acid sequences of non-carbapenemase *P. aeruginosa* strains (*n* = 6) that did not produce an ESBL identified two strains producing the PDC-322, which harbored the mutation G183D, conferring resistance to C/T [33, 34]. Additionally, four strains producing PDC-157, PDC-240, PDC-407, and PDC-565 were identified, and no substitution known to be responsible resistance to C/T was identified among those AmpC sequences.

### Susceptibility to FDC against CRP clinical isolates

A high susceptibility rate was evidenced with FDC (91%) when testing all CRP isolates. Interestingly, a susceptibility rate to FDC of 80% was found when testing the 49 carbapenemase-producing isolates. When considering only the MBL-producing isolates (*n* = 47), the susceptibility rate for FDC was evaluated at 79%, with a higher proportion of VIM-like and IMP-like producers being susceptible in comparison to the NDM-1 producers. When considering non-carbapenemase producing isolates only, the susceptibility rate to FDC reached 95%. Hence, whatever the sub-categorization in term of carbapenem resistance mechanism, FDC exhibited the highest susceptibility rate among the different last-resort therapeutical options tested (Fig. 1).

**Figure 1 Distribution of MIC values determined for cefiderocol against carbapenem-resistant *Pseudomonas* spp. *Pseudomonas *spp. isolates**.

Resistance to FDC was observed for only 16 isolates, including 10 isolates producing MBLs, namely NDM-1 (*n* = 8), VIM-2 (*n* = 1) or VIM-5 (*n* = 1), two isolates producing an ESBLs (namely GES-7), and four isolates for which neither production of a carbapenemase nor of an ESBL could be identified.

Of note, the susceptibility rate of colistin was found to be high (above 95%), in all categories of CRP clinical isolates tested here.

### Whole-sequencing analysis for FDC-resistant isolates in comparison with FDC-susceptible isolates

WGS of the 16 FDC-resistant and a set of 16 FDC-susceptible isolates identified several interesting genetic features (Table 2). Regarding acquired β-lactamase content, the following enzymes were more frequently identified among FDC-resistant isolates; NDM-1 (*n* = 8 vs. 3), GES-7 (*n* = 2 vs. 0), VEB-14 (*n* = 1 vs. 0). When considering the nature of the intrinsic AmpC ß-lactamase, the PDC-16 (*n* = 7) variant was the most commonly identified among FDC-resistant isolates, although this variant was not identified among the FDC-susceptible isolates.

In term of strain background, a total of eight different STs were identified, with ST773 being the most prevalent. Noteworthy, ST773 strains were all part of the FDC-resistant isolates, and this clonal background was associated to the production of NDM-1, although ST111 strains were associated to the production of GES-7 or VIM-like enzymes, and ST111 strains to VIM-like enzymes.

**Table 2:** MIC values and genetic features associated to cefiderocol susceptibility/resistance among *P. aeruginosa* clinical isolates

Strain	Sample origin and date (MM/YY)	FDC^a^ MIC value (mg/L)	β-Lactamase content^b^	ST-type	TonB-dependent receptor proteins^c^					Iron uptake system proteins^c^		Porin protein^c^	Efflux regulators proteins^c^	
					piuA/piuD	piuB	pirA	pirR	pirS	fecA	fecI	oprD	mexR	nalD
PA36	Bern 01.2022	16	OXA-851 (c), PDC-322 (c)	645	Q34H	A573T H604N	A370T	Truncated	Q77R N126S G360D	V95A H363R	WT	disrupted	WT	Q213_P265Ins
PA48	Zürich 01.2022	4	PDC-35 (c), GES-7 (c), OXA-488 (c)	235	T411I*	A609V	A370T S20N T235I	WT	WT	V95A A113V T288I T339A G358S T359A H363R R571Q	WT	disrupted	V126E	V151_M158del
PA82	Luzern 01.2022	4	OXA-488 (c), PDC-34 (c)	253	Q34H	WT	A370T	WT	WT	V95A S2F T339A G358S T359A H363R R571Q	WT	disrupted	V126E	Truncated
PA54	Luzern 03.2022	4	PDC-35 (c), GES-7 (c), OXA-488 (c)	235	T411I*	A609V	A370T S20N T235I	WT	WT	V95A A113V T288I T339A G358S T359A H363R R571Q	WT	disrupted	V126E	V151_M158del
PA76	Geneva 03.2022	4	OXA-395, PDC-16, NDM-1 (p)	773	Q34H	G382S A609V	A370T	WT	N126S	V95A T298A T339A G358S T359A H363R R571Q	WT	disrupted	V126E	WT
PA08	Bern 05.2022	64	OXA-1032 (c), PDC-22 (c)	667	Q34H Truncated	NA	A370T	F2L E69D	WT	V95A V212A G358S T359A T339A H363R R571Q	WT	disrupted	Truncated	WT
PA72	Luzern 07.2022	4	OXA-395 (c), PDC-16 (c), NDM-1 (p)	773	Q34H	G382S A609V	A370T	WT	N126S	V95A T298A T339A G358S T359A H363R R571Q	WT	disrupted	V126E	WT
PA10	Bern 07.2022	4	OXA-395 (c), PDC-16 (c), NDM-1 (p)	773	Q34H	G382S A609V	A370T	WT	N126S	V95A T298A T339A G358S T359A H363R R571Q	WT	disrupted	V126E	WT
PA136	Zürich 07.2022	4	PDC-3 (c), OXA-395 (c), VIM-2 (p)	111	WT *	P51S A143V A573T H604N	A370T	A52G	Q77R N126S L213F G360D	A342V H363R	ND	disrupted	V126E	WT
PA58	Basel Land 08.2022	4	OXA-395 (c), PDC-30 (c)	207	Q34H	F165L D574N A594V H604N	A370T K590T D608G	F2L	N126S	V95A T339A G358S T359A H363R R571Q	WT	disrupted	V126E	R13C
PA12	Zürich 09.2022	4	VIM-5 (c), OXA-846 (c), PDC-11 (c), VEB-14 (p), OXA-10 (p)	357	WT *	F165L L197F A351V A609V	Y2S A370T	WT	A304V	V95A T339A G358S T359A H363R R571Q	WT	disrupted	V126E	Truncated
PA69	Tessin 11.2022	4	OXA-395 (c), PDC-16 (c), NDM-1 (p)	773	Q34H	G382S A609V	A370T	WT	N126S	V95A T298A T339A G358S T359A H363R R571Q	WT	disrupted	V126E	WT
PA72	Luzern 11.2022	4	OXA-395 (c), PDC-16 (c), NDM-1 (p)	773	Q34H	G382S A609V	A370T	WT	N126S	V95A T298A T339A G358S T359A H363R R571Q	WT	disrupted	V126E	WT
PA13	Luzern 12.2022	8	PDC-16 (c), OXA-395 (c), NDM-1 (p)	773	Q34H	G382S A609V	A370T	WT	N126S	V95A T298A T339A G358S T359A H363R R571Q	WT	disrupted	V126E	WT
PA35	Zürich 12.2022	4	PDC-16 (c), OXA-395 (c), NDM-1 (p)	773	Q34H	G382S A609V	A370T	WT	N126S	V95A T298A T339A G358S T359A H363R R571Q	WT	disrupted	V126E	WT
PA53	Basel Land 12.2022	4	PDC-16 (c), OXA-395 (c), NDM-1 (p)	773	Q34H	G382S A609V	A370T	WT	N126S	V95A T298A T339A G358S T359A H363R R571Q	WT	disrupted	V126E	WT
PA23	Zürich 05.2022	0.25	OXA-395 (c), PDC-3 (c), OXA-9 (p), VIM-4 (p)	111	WT	P51S A143V A573T H604N	A370T	A52G	Q77R N126S V180I L213F G360D	A342V	NA	disrupted	V126E	WT
PA24	Zürich 05.2022	0.25	PDC-35 (c), OXA-488 (c), VIM-2 (p)	235	T411I*	A609V	S20N T235I A370T	WT	WT	V95A A113V T288I T339A G358S T359A R571Q	WT	T103S K115T F170L P186G V189T R310E A315G G425A	V126E	WT
PA65	Bellinzona 06.2022	0.25	OXA-488 (c), PDC-30 (c), VIM-2 (p)	671	K729Q*	S454C Q465H A573T	V128A A370T	WT	Q77R N126S	V95A A298V T339A G358S T359A R571Q A714V	WT	S57E S59R V127L P186G V189T I210A E230K S240T N262T T276A K296Q Q301E R310E A315G L347M V372_G383Ins S403A Q426E	WT	WT
PA70	Zürich 07.2022	0.25	PDC-35 (c), OXA-488 (c), VIM-5 (p)	235	T411I*	A609V	S20N T235I A370T	WT	WT	V95A A113V T288I T339A G358S T359A R571Q	WT	T103S K115T F170L P186G V189T R310E A315G G425A	V126E	WT
PA90	Buchs 07.2022	0.25	OXA-905 (c), PDC-8 (c), OXA-10 (p), VIM-2 (p)	395	WT*	S311N A384V D574N A594V H604N E806G	A370T	A52G	Q77R N126S V180I E272D Q274R D328A	WT	WT	D43N S57E S59R I210A E230K S240T N262T A267S K296Q Q301E R310G V359L V372_G383Ins	WT	WT
PA16	Luzern 07.2022	0.5	PDC-12 (c), OXA-488 (c), OXA-10 (c), IMP-1 (c)	1047	Q34H	E785G	A370T	WT	WT	V95A G358S T359A R571Q	WT	T103S K115T F170L P186G V189T R310E A315G G425A	V126E	NA
PA38	Zürich 07.2022	0.25	PDC-35 (c), OXA-488 (c), VIM-2 (c), NDM-1 (c)	235	T411I*	A609V	S20N T235I A370T	WT	WT	V95A A113V T288I T339A G358S T359A R571Q	WT	disrupted	V126E	WT
PA89	Sion 08.2022	0.25	OXA-395 (c), PDC-3 (c), OXA-9 (p), VIM-4 (p)	111	WT*	P51S A143V A573T H604N	A370T	A52G	Q77R N126S V180I L213F G360D	A342V	NA	disrupted	V126E	WT
PA03	Liebefeld 08.2022	0.25	OXA-395 (c), PDC-3 (c), VIM-2 (p)	111	WT*	P51S A143V A573T H604N	A370T	A52G	Q77R N126S V180I L213F G360D	A342V	NA	disrupted	V126E	WT
PA18	Bellinzona 08.2022	0.5	PDC-19a (c), OXA-488 (c), NDM-1 (p)	308	Q34H P79L	F165L G844R	A370T	WT	N126S S260P	V95A Q122P T339A G358S T359A R571Q A714V	WT	T103S K115T F170L P186G V189T R310E A315G G425A	V126E	Truncated
PA40	Geneva 08.2022	0.5	PDC-3 (c), OXA-395 (c), VIM-2 (p)	111	Q34H	P51S A143V A573T H604N	A370T	A52G	Q77R N126S V180I L213F G360D	A342V	NA	disrupted	V126E	WT
PA29	Bern 09.2022	2	OXA-488 (c), PDC-46 (c)	1917	Q34H	F165L D574N A594V H604N	A370T K590T D608G	Truncated F2L	N126S V180I S260P	V95A F155L T339A G358S T359A R571Q	WT	disrupted	V126E	WT
PA98	Bern 07.2022	2	OXA-396 (c), PDC-3 (c), GES-1 (c), NDM-1 (c)	654	WT*	T301I A573T E598D H604N	P275S A370T G578E	A52G	Q77R N126S V180I D328A	A27T V95A A120T	WT	V127L P186G V189T I210A E230K S240T N262T T276A K296Q Q301E R310E G312R A315G G316D L347M V372_G383Ins S403A Q426E	WT	L153Q
PA47	Bern 10.2022	0.5	OXA-488 (c), PDC-158 (c), VIM-2 (p)	2644	K729Q*	WT	A370T R549S T683I	WT	N126S S260P	V95A T339A G358S T359A R571Q	G13D	disrupted	V126E	WT
PA57	Zürich 10.2022	0.25	OXA-10 (c), OXA-488 (c), PDC-12 (c), IMP-1 (c)	1047	Q34H	E785G	A370T	WT	WT	V95A G358S T359A R571Q	WT	T103S K115T F170L P186G V189T R310E A315G G425A	V126E	NA
PA176	Sion 11.2022	0.25	OXA-396 (c), PDC-3 (c), GES-5 (c)	654	WT*	T301I A573T E598D H604N	P275S A370T G578E	A52G	Q77R N126S V180I D328A	A27T V95A A120T	WT	V127L P186G V189T I210A E230K S240T N262T T276A K296Q Q301E R310E G312R G314D A315G L347M V372_G383Ins S403A Q426E	WT	WT

(a) FDC. Cefiderocol (b) (c), chromosomally-encoded ß-lactamase; (p), plasmid-mediated ß-lactamase; * substitution found in the *piuD* gene, (c) WT, wild-type gene; NA. data not available

When considering the non-enzymatic genetic features, several substitutions were identified in TonB-dependent receptor proteins among the FDC-resistant isolates, namely PiuA (Q34H), PiuD (T411I), PiuB (i.e. A609V, G382S, H604N), PirA (i.e. A370T, S20N, T235I), PirR (F2L, A52G, E69D), PirS (i.e. N126S, Q77R, G360D), and FecA (i.e. V95A, T339A, G358S, T359A, H363R, R571Q). These proteins are all involved in iron transport systems and might be affecting the susceptibility to FDC. However, most of those substitutions were also identified among FDC-susceptible isolates. Careful analysis identified only few substitutions being exclusively found among FDC-resistant isolates, namely PiuB (G382S, L197F, A351V), PirA (Y2S), PirR (E69D), PirS (A304V), FecA (H363R, S2F, V212A). Interestingly, we observed that Arg363 in FecA was constantly present only among FDC-resistant isolates, but never among FDC-susceptible isolates. In addition, all FDC-resistant isolates had a disrupted OprD protein sequence, which is known to significantly and negatively impact the permeability of the bacterial cell with respect to imipenem penetration. Finally, substitutions, deletions and insertions were found within efflux regulatory proteins, such as MexR (V126E, or truncation) or NalD (truncation, V151_M158del, Q213_P265Ins), likely contributing to the upregulation of the main efflux pump MexAB-OprM in those FDC-resistant isolates.

## Discussion

Our study highlighted that the β-lactam-based therapeutics exhibiting the optimal in-vitro activity against CRP collected across Switzerland was FDC, regardless of the carbapenem resistance mechanisms. Interestingly, our data indicated that FDC exhibited a susceptibility rate exceeding 80% across all strain subgroups, including an activity of 79% among MBL-producing isolates. This therapeutical option was particularly effective against VIM-producing *Pseudomonas* spp. isolates, which are the most prevalent MBL-producing *P. aeruginosa* in Europe, and usually leave very few therapeutic alternatives [6, 35, 36].

Although C/T and IPR were not effective therapeutic options for carbapenemase producers, both combinations showed high susceptibility rates (over 82%) against non-carbapenemase CRP isolates, corresponding to the most common phenotype among CRP isolates worldwide [6]. Those data are in line with previous work conducted in Canada or Spain, reporting FDC as the most effective in-vitro option against multi-drug or extensively-drug resistant *P. aeruginosa* isolates [37–40], even if some other reports showed that IPR could be an alternative for isolates showing reduced susceptibility to FDC [41]. Interestingly, we showed here that half of the FDC-resistant isolates that had been collected from different parts of Switzerland corresponded to a single genetic background, being the *P. aeruginosa* ST773 producing NDM-1, therefore highlighting a worrying dissemination of a multidrug-resistant clone. This clonal dissemination has already been described in Europe related to the Ukraine patients [42]. Noteworthy, among the sixteen different STs identified in both FDC-resistant and susceptible isolates, four FDC-resistant isolates distributed in three STs (111, 235, and 357) are considered members of the worldwide Top10 high-risk clones [6]. These findings are similar to those reported in a previous study, analyzing PA-MBL isolates collected from 2022 to 2023 in Switzerland, with ST111, ST773 and ST1047 dominating the country [36]. Our findings further highlight that multiple modifications in iron transporter systems, particularly the H363R substitution in FecA operon, being constantly and specifically found in FDC-resistant isolates, associated to efflux system upregulation and porin deficiency, constitute the main source of FDC resistance in *Pseudomonas* spp. Most of mutations found in this study were previously reported [10–13, 43–45]. Nevertheless, FDC overall showed excellent activity against most CRP Swiss isolates, as well as colistin.

When specifically considering the non-carbapenemase CRP isolates, representing the most common feature among multidrug-resistant *P. aeruginosa* [6], the novel commercially-available BL/BLI combinations C/T and IPR were interesting therapeutical options, superior to CZA and MEV. The efficacy of C/T can be attributed to the fact that ceftolozane is one of the most active antipseudomonal cephalosporins, targeting multiple penicillin-binding proteins and evading the hydrolytic activities of the majority of AmpC β-lactamases and class D β-lactamases. In contrast, tazobactam, which does not inhibit class C enzymes, has been demonstrated to significantly inhibit the majority of class A extended-spectrum β-lactamases, potentially including ceftolozane within their hydrolytic spectrum [5, 6, 46, 47]. Previous studies already showed that the C/T combination is highly active against ceftazidime-resistant or carbapenem-resistant *P. aeruginosa*, but this activity decreased against multidrug-resistant or XDR *P. aeruginosa* isolates, as well as against MBL producers [41, 44, 45, 48]. The relatively high resistance rate observed for C/T may be partially explained by the nature of the collection tested here, including only carbapenem-resistant isolates. Furthermore, some isolates were found to produce ESBLs and/or specific AmpC variants known to confer resistance to C/T. Altogether, those different features may have contributed to the observed resistance to C/T among non-carbapenemase producing CRPs.

Our data also showed that IPR overall possesses a relatively poor activity against CRP, being however significantly better when considering non-carbapenemase producing isolates only. These results are in agreement with previous studies [49, 50] and can likely be explained by the effective inhibition of the natural AmpC (PDC) of *Pseudomonas* spp. by relebactam, restoring imipenem activity when considering imipenem non-susceptible *Pseudomonas* spp. isolates [13, 27, 47, 51]. In line with others studies, CZA and MEV were relatively less efficient [52].

## Conclusion

In this study, FDC showed the best in-vitro activity against CRP circulating in Switzerland in 2022 especially against MBL producers. Although the novel BL/BLI combinations MEV, CZA, C/T and IPR are poorly effective against carbapenemase producers, mainly corresponding to MBL producers, they showed a significant in-vitro activity against the non-carbapenemase producers, C/T and IPR being the most active with susceptibilities rates of 83% and 82%, respectively. Finally, the analysis of FDC-resistant isolates highlighted a specific high-risk clone ST773 NDM-1-producing *P. aeruginosa* widely distributed in Switzerland, being worryingly resistant to all BL/BLI combinations tested in this study. Even though we believe our collection might reflect the overall actual European epidemiology of CRP isolates, we acknowledge it would be risky to extrapolate these findings to other contexts, and therefore other similar epidemiological studies will be interesting to conduct all over Europe to establish the optimal therapeutics.

## Electronic supplementary material

Below is the link to the electronic supplementary material.

Supplementary Material 1

Supplementary Material 2

## Data Availability

Data presented in this manuscript can be available upon request.
