# Supplementary figures and images for: In-vitro activity of the novel β-lactam/β-lactamase inhibitor combinations and cefiderocol against carbapenem-resistant Pseudomonas spp. clinical isolates collected in Switzerland in 2022
Source: Eur J Clin Microbiol Infect Dis. 2024 Dec 20;44(3):571–85. doi: 10.1007/s10096-024-04994-6 (PMC11880081; doi:10.1007/s10096-024-04994-6)

Susceptible isolates

*EUCAST breakpoint*

Resistant isolates

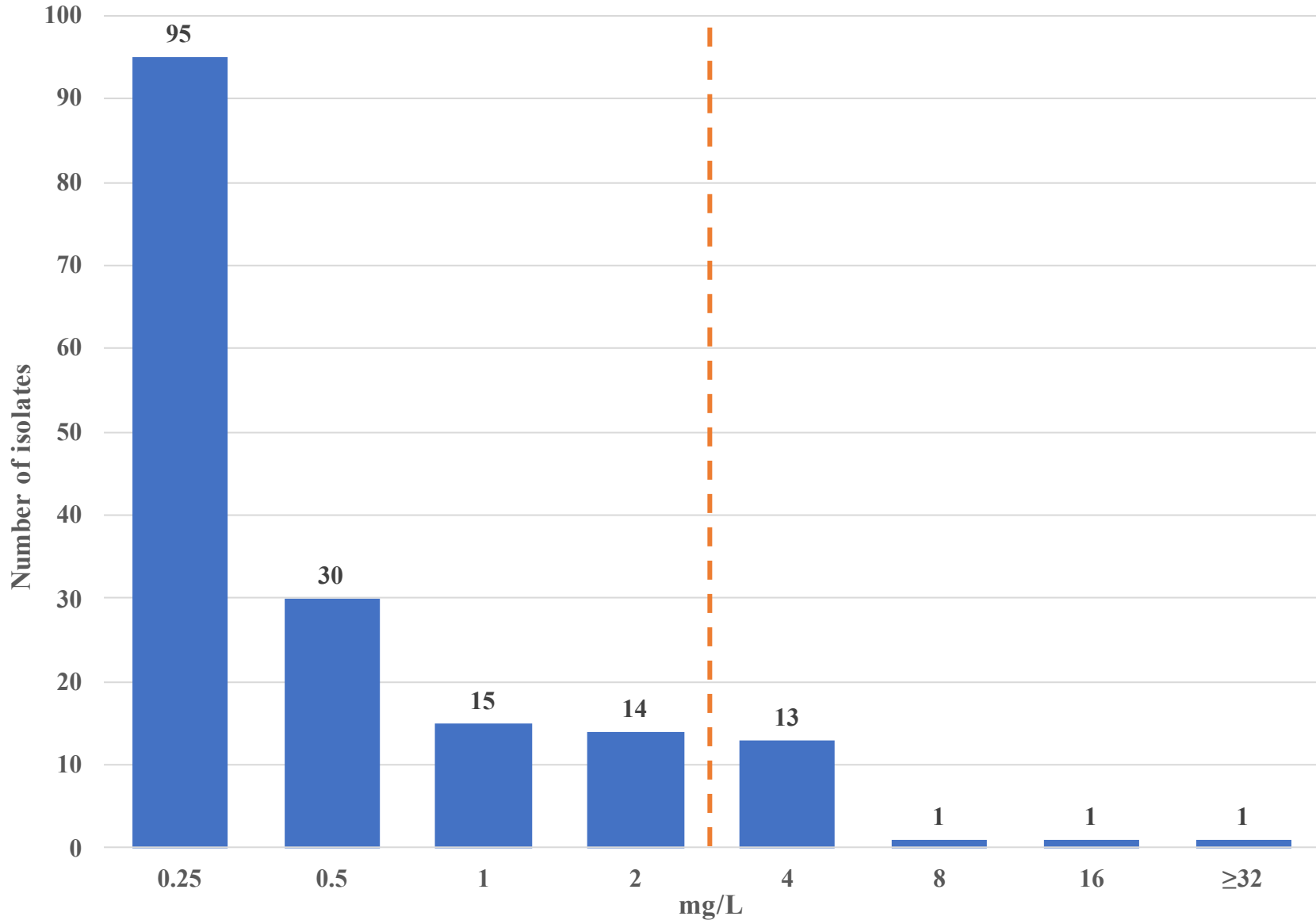

Supplement: Supplementary file 2 — Supplementary Material 2 [file 10096_2024_4994_MOESM2_ESM.pdf]
